# Effect of alendronate sodium plus vitamin D_3_ tablets on knee joint structure and osteoarthritis pain: a multi-center, randomized, double-blind, placebo-controlled study protocol

**DOI:** 10.1186/s12891-022-05521-4

**Published:** 2022-06-17

**Authors:** Hui-ming Peng, Xi-sheng Weng, Ye Li, Bin Feng, Wenwei Qian, Dao-zhang Cai, Chang Zhao, Zhen-jun Yao, Yi Yang, Chi Zhang, Shengcheng Wan

**Affiliations:** 1 Department of Orthopaedic Surgery, State Key Laboratory of Complex Severe and Rare Diseases, Peking Union Medical College Hospital, Chinese Academy of Medical Science and Peking Union Medical College, No.1, Shuaifuyuan Wangfujing, Dongcheng District, Beijing, 100730 China; 2 Department of Joint Surgery, Center for Orthopaedic Surgery, The Third Affiliated Hospital of Southern Medical University, Guangzhou, 510630 China; 3 Department of Orthopaedic Surgery, Zhongshan Hospital Fudan University, Shanghai, 200032 China

**Keywords:** osteoarthritis, pain, alendronate, vitamin D3, trial

## Abstract

**Background:**

Osteoarthritis (OA) is a major cause of pain and disability worldwide. Despite the relatively high burden of the disease, the currently available non-surgical treatment options are directed towards symptomatic relief. Therefore, we propose the use of alendronate as a disease modifying agent to help slow and prevent OA. In addition, this study will utilize Whole-Organ Magnetic Resonance Imaging Score (WORMS) to evaluate the structural integrity of cartilage in the study population. High-quality evidence, limited to a few well-conducted randomized trials, highlights contradictory results on the effect of bisphosphonates on knee function and progression of OA. Therefore, a placebo-controlled, randomized trial is needed to evaluate the combined effect of alendronate and vit D on the structure of cartilage utilizing the WORMS score and its ability to treat knee pain in OA patients.

**Methods:**

This multicenter, randomized, double-blinded, placebo-controlled study will evaluate the efficacy and safety of alendronate in early OA. Patients will undergo a 1:1 double-blinded randomization to receive a one-year course of either alendronate sodium vitamin D_3_ or placebo. The primary outcome is to compare WORMS score of knee joint at 6 and 12 months between both groups. Secondary endpoints will include WORMS score at 24 months, knee pain, radiographic progression of OA, severity of OA, quality of life, and serum inflammatory biomarkers at different assessment timepoints. To detect a 2.2% difference in cartilage loss between both groups with power of 80%, a sample size of 60 (30 per group) is proposed.

**Discussion:**

This trial will give helpful and high-quality evidence regarding the potential therapeutic role of alendronate sodium vitamin D3, as compared to placebo, in the management of patients with knee OA regarding its role on cartilage loss, radiographic progression of OA, severity of OA, knee pain, quality of life, and inflammatory biomarkers. If proven effective, this intervention would be a great option for providing beneficial outcomes with a reduced cost in this patient population.

**Trial registration:**

This trial was registered on clinicaltrials.gov (registration number: NCT04739592).

**Supplementary Information:**

The online version contains supplementary material available at 10.1186/s12891-022-05521-4.

## Background

Osteoarthritis (OA) is a major cause of pain, functional limitations, and disability worldwide [1]. The hip and knee joints are the most common sites of OA-associated disability worldwide [2]. The causes of pain and mechanical dysfunction in osteoarthritis (OA) remain unclear, and previous research have suggested multifactorial causes [3–6]. Injury to one component of the joint leads to a cascade of injuries that lead to further joint dysfunction and clinical manifestations of OA. Currently, only a few features, such as joint space narrowing and osteophytes can be detected on standard radiographs, and determining the severity of disease is limited. Therefore, a broader imaging assessment scale is needed to correctly evaluate joint structural integrity and its role in the symptomatic manifestations of OA.

The Whole-Organ Magnetic Resonance Imaging Score (WORMS) is a good semi-quantitative tool for the study of knee osteoarthritis (KOA). The WORMS score includes cartilage signal and morphology, subchondral bone marrow abnormalities, subchondral cysts, subchondral bone wear, marginal osteophytes, and other features which can comprehensively analyze the morphology and structure of knee OA. The WORMS scoring system requires magnetic resonance imaging (MRI), which not only has no ionizing radiation, but also avoids the morphological aberration, amplification, and superposition seen with computer tomography. More importantly, MRI is well suited for evaluating joint tissues such as cartilage, meniscus, and ligaments, giving it great potential as an imaging tool for the entire joint. The knee MRI scans will be taken using a 1.5-T whole-body MRI unit with a transmit-receive coil. The same scanning unit will be used for all participants in the study center. The sequence and parameters of the used MRI unit is provided in (Supplementary Table 1).

For osteoarthritis (OA), current treatments focus on symptomatic relief, but many patients do not achieve adequate pain control [7]. In addition, despite the high prevalence of OA, there are currently no approved disease-modifying drugs (DMOADs) for OA that would help prevent the joint damage seen along the course of the disease. Therefore, the development of a novel and effective treatment is necessary.

Bisphosphonates are a class of drugs commonly used to treat osteoporosis due to their ability to inhibit osteoclast-mediated bone loss. Several studies have reported favorable results of bisphosphonates as a potential treatment option in osteoarthritis. In animal studies, bisphosphonates (alendronate, tiludronate) have demonstrated the ability to slow the progression of OA-related structural damage [8–10]. Similarly, a clinical study found that the use of bisphosphonates (including ibandronate, pamidronate, risedronate, and zoledronate) to be associated with a 26% reduction in the risk of knee replacement in older women [11].

In 2017, a meta-analysis was conducted to compare the efficacy of bisphosphonates to placebo in patients with knee OA in terms of knee pain, radiographic progression, and adverse events [12]. Based on the analysis of seven randomized controlled trials, no statistically significant differences were noted between both groups regarding knee pain and function, radiographic progression, or adverse events. However, no subgroups analyses were reported regarding the type of bisphosphonate, dose or route, assessment timepoint, or measurement scale.

During the first 3 years of observation, alendronate which was most commonly used (> 60%) in the Osteoarthritis Initiative (OAI) [13] showed a reduction in knee pain for the women taking the medication. Additionally, joint space narrowing (JSN) decreased over time in bisphosphonate users. Alendronate has also been found to delay progression of spinal osteophytes and disc space narrowing suggesting it may be able to slow the progression of spinal osteoarthritis [14].

High-quality evidence, limited to a few well-conducted randomized trials, highlights contradictory results on the effect of bisphosphonates (of different doses and administration routes) on knee function and disease progression in patients with knee OA [15–17]. Furthermore, another ongoing multicenter, randomized, double-blinded, placebo-controlled trial will compare the efficacy of annual infusions of zoledronate to placebo in terms of knee joint structural changes and knee pain in patients with knee OA [18]; however, the results are expected in 2022–2023. Therefore, and based on the above-mentioned observations, we are willing to conduct this randomized, placebo-controlled trial to determine the effect of combined alendronate sodium with vit D on the knee joint structure, knee pain, radiographic grade of OA, quality of life (QOL), and severity of OA in patients with knee OA and MRI evidence of bone marrow edema-like lesions since they show greater response to bisphosphonates [19].

### Trial objectives

This designed randomized controlled trial will explore the efficacy of alendronate sodium vitamin D_3_ compared to placebo on the improvement of joint structure and pain in patients with knee osteoarthritis. Our primary hypothesis is that there will be a significant improvement in the joint structure, as measured by the WORMS scale, in favor of alendronate at each assessment timepoint. Our secondary hypothesis is that there will be a significant improvement in knee pain, severity of OA, and quality of life, as well as a significant reduction in the level of inflammatory biomarkers, among patients assigned to the alendronate sodium vitamin D3 as compared to those given placebo.

## Methods/design

### Study design and setting

This is a multicenter, randomized, double-blinded, placebo-controlled study. Upon obtaining Institutional Review Board (IRB)- Ethics Committee’s approval (from each corresponding included center), recruitment of patients will begin. The study is scheduled to start on May 1, 2021, with a completion date on December 31, 2022. Patients will undergo a 1:1 double-blinded randomization to receive a one-year course of alendronate sodium vitamin D_3_ tablets (Gubangjia, The CSPC Ouyi Pharmaceutical Co., Ltd. Shijiazhuang, China) or placebo. The use of Tylenol for pain control will be permitted during the trial; however, it will be monitored and reported for future analysis. In addition, Vitamin D and calcium supplementation will be allowed for usage by study participants. The use of unmarketed drugs or other investigational drugs will be prohibited during the study period; otherwise, such patients will be excluded. Other bisphosphonates (such as zoledronic acid, risedronic acid, etc.) will be prohibited during the study period. Non-steroidal anti-inflammatory drugs and other topical drugs with analgesic effects will be prohibited during the study period. This study will be carried out in three domestic hospitals (Peking Union Medical College Hospital, Zhongshan Hospital of Fudan University, and The Third Affiliated Hospital of Southern Medical University) with a follow-up period of 2 years.

### Eligibility criteria

#### Inclusion criteria

Patients will be eligible for inclusion if they are consistent will all of the following criteria: (1) adult patients (50–75 years of age) with significant knee pain (defined as pain score ≥ 40 mm on the 100mmVAS scale), (2) having a diagnosis of knee osteoarthritis per the diagnostic criteria of the American College of Rheumatology (ACR) and the Chinese Orthopedic Association, and (3) having a radiographic Kellgren-Lawrence grade I or II knee OA and MRI findings of bone marrow edema-like lesions. The inclusion of bone marrow edema-like lesions (identified by high bone turnover through MRI) is because they are associated with greater response to bisphosphonates [19].

#### Exclusion criteria

Patients will be excluded if they have one of the following criteria: (1) having any forms of active arthritis (such as rheumatoid arthritis or other inflammatory arthritis), (2) users of non-steroidal anti-inflammatory drugs (NSAIDs) and central analgesics (such as opioids) within two weeks or other study drug or device within 30 days before randomization or within the drug half-life (whichever is longer), (3) evidence of abnormal liver function tests (ALT or AST; at least a 1.5-fold increase above the upper limit of normal), (4) having renal dysfunction, osteomalacia, or creatinine clearance < 35 mL/min, (5) having serious heart disease, endocrine, digestive, mental, nervous system diseases, cancer, or serious illnesses with a life expectancy of less than two years, (6) pregnancy or breast-feeding, (7) having active ulcers, history of upper gastrointestinal bleeding, or esophageal motility disorders, (8) having fracture within 6 months of study initiation, (9) tobacco addiction or alcohol abuse, (10) having allergy or contraindication to study medications, (11) contraindications to MRI or X-rays exposure, (12) planned joint replacement within the study period, or (13) having poor dental health or scheduled dental surgery before or during the study period.

### Patient recruitment

According to the principle of fair distribution of burden and benefit, relevant information will be released through a participant recruitment announcement → prospective participants will read the study introduction → informed consent will be obtained from eligible individuals → selected participants will then undergo randomization to receive the treatment or placebo.

### Randomization and blinding

Randomization will be performed using a variable-sized block (sizes of 4, 8, and 12), computer-generated randomization procedure to ensure equal number of participants within each treatment arm. The execution of block randomization will be done by the SAS software, where a different block will be implemented each time the value within the parenthesis of the ranuni function of the macro set reaches zero. The assignments will be blinded to both the investigators and study participants and concealed within sealed, fully opaque envelops. Of note, emergency unblinding will only be allowed in critical situations where the safety of included participants is affected.

Allocation concealment will be ensured with the use of sequentially numbered, opaque, sealed envelopes within containers. There will be a coordinating center for this particular step and a particular study personnel will carry out the allocation of patients.

### Outcome measures

#### Primary outcome

Our primary outcome is to determine the improvement in knee structure following each intervention using an MRI-based scale named the Whole-Organ Magnetic Resonance Imaging Score (WORMS) scale at 6 and 12 months after treatment allocation [20]. The WORMS scale is designed to determine the status of the knee joint in patients with OA in respect to fourteen independent articular features. Five out of the 14 features are related to the articular surfaces, and they include: (1) cartilage signal and morphology, (2) subarticular bone marrow abnormality, (3) subarticular cysts, (4) subarticular bone attrition, and (5) marginal osteophytes. In addition, all of these features are further evaluated in 15 different regions divided by anatomical landmarks in a fully-extended knee (Supplementary Fig. 1). For the purposes of our research, we will focus on cartilage signal and morphology. This feature is scored based on only 14 articular surface regions (excluding region S), with a score ranging from 0 to 6 on an 8-point scale (Supplementary Fig. 2). The WORMS scoring system for cartilage includes 0 = normal cartilage thickness and signal, 1 = normal cartilage thickness but with increased signal, 2 = a partial-thickness defect of < 1 cm in the greatest width, 2.5 = a full-thickness focal defect of < 1 cm in the greatest width, 3 = multiple regions having partial-thickness defects alternating with regions of normal thickness or defects > 1 cm, 4 = diffuse partial-thickness defects, 5 = multiple regions of full-thickness defects or defects > 1 cm, and 6 = diffuse full-thickness defects, with an overall score ranging from 0 to 84.

#### Secondary outcomes

We have seven pre-identified secondary outcomes: WORMS score (at 24 months), pain intensity (at 2, 4, 8, and 12 weeks after enrollment), quality of life (QOL), bone density, risk of knee replacement, severity of knee OA, and estimation of inflammatory biomarkers.

1. The structure of knee joint will be measured using the WORMS scale at 24 months after treatment.
2. Knee pain will be measured in both groups at 2, 4, 8, and 12 weeks after enrollment using the 100-mm visual analogue scale (VAS), which has already been shown to be valid and reliable in measuring pain [21]. Overall ratings range from 0 to 100 mm, where 0–4 mm indicate no pain, 5–44 mm indicate mild pain, 45–74 mm indicate moderate pain, and 75–100 mm indicate severe pain.
3. Quality of life in both groups will be assessed at 3, 6, and 12 months after enrollment using the previously validated Chinese version of the SF-36 questionnaire [22], which is used to measure health status based on eight categories: (1) physical activity, (2) social activity, (3) physical role, (4) energy and emotional status, (5) mental health, (6) vitality, (7) body pain, and (8) general health. The overall score ranges from 0–100, with higher scores reflecting better health-related QoL [23]. This version of the SF-36 questionnaire shows comparable validity and reliability to the original version.
4. Bone density of the lumbar spine and the hip and knee joints will be carried out using dual-energy x-ray absorptiometry (DXA) at 12 and 24 months after enrollment. Bone density data will be obtained using a Prodigy DXA scanner (GE Healthcare, Madison, USA) and the obtained data will be analyzed according to the manufacturer’s software. The lumbar spine (L1-L4) and the hip (particularly the femur neck and total hip) will be scanned in the supine position using posteroanterior projections.
5. The risk of knee replacement will be evaluated based on a set of baseline and clinical variables and then incorporated into a multivariable logistic regression model to identify significant predictors of knee replacement in patients with OA.
6. Severity of OA will be measured at 12 and 24 months after enrollment using the Kellgren-Lawrence grading scale [24]. The scores of this grading scale range 0 to 4, where 0 indicates no features of OA, 1 indicates questionable osteophytes, 2 indicates definite osteophytes without narrowing in the joint space, 3 indicates definite osteophytes with moderate narrowing, and 4 indicates definite osteophytes with severe narrowing.
7. The serum level of the following inflammatory biomarkers will be measured at 3, 6, and 12 months after enrollment: interleukin (IL)-6, tumor necrotic factor (TNF)-alpha, erythrocyte sedimentation rate (ESR), and C-reactive protein (CRP).

### Safety analysis

Based on the SAS dataset, Cox proportional risk model will be used to calculate and compare the hazard ratios (HR values) between the two groups for most of the safety endpoints. Poisson regression or negative binomial regression will be used to better fit the rare events. Adverse events and serious adverse events will be listed separately and analyzed in summary. In each treatment group, the number and percentage of adverse events will be summarized by tissue and organ category. All deaths and serious adverse events will be described in detail using case narrative style.

### Sample size calculation

Based on a previous observation, the mean annual loss of the medial tibial cartilage volumes in knee OA patients is 4.5% [25]. The monthly intake of vitamin D (50,000 IU) would result in a serum vitamin D3 level > 60 nmol/L [26], and this, in turn, has been reported to correlate with a 2.2% annual absolute reduction in cartilage loss [27]. This can be translated into a risk reduction of 22% for knee replacement over a period of 2 years [28]. Our sample size calculation was conducted based on Cohen’s formula [29]. With a power level of 80% and alpha level of 0.05, 60 participants (30 in each group) were required to detect a 2.2% between-group difference in terms of medial tibial cartilage loss (WORMS scale) during the second assessment time-point (12 months). Losses to follow-up were not considered in our initial sample size calculation (*NCT04739592*); however, given the fact that both interventions will be administered for a year and patients will be followed up for 2 years after enrollment, we anticipate that around 10% will be lost to follow-up each year, so we increased our final sample size to 220 participants (110 in each group).

### Statistical analysis

Retrieved data from each patient in both groups will be recorded at baseline and at each follow-up assessment period using Excel, which will then be entered into a software for analysis after the final assessment point has been met. All statistical analysis will be conducted by a third-party (not involved with our research in any way) using the Statistical Analysis System (SAS) Software for Windows (version 9.4, *SAS institute, USA*).

Baseline demographic and clinical variables will be presented in the form of number and percentages for categorical variables, while continuous variables will be presented as means and standard deviations (SD) or median and range if the data were normal or skewed, respectively.

Our primary outcome will be analyzed with the intention-to-treat (ITT) analysis and reported as the mean difference (MD) and corresponding 95% confidence interval (CI) in cartilage-WORMS score between the intervention and control groups using Student’s t-test. Secondary outcomes will then be analyzed at the final assessment. Pain will be analyzed as a categorical outcome using the chi-square test, while the differences in continuous determinants of pain (baseline variables) will analyzed using MANOVA test. The QoL will be analyzed and reported as the mean difference and its corresponding 95% CI between both interventions using t-test. Bone density and osteoporosis severity (measured by the Kellgren-Lawrence scale) will be analyzed as categorical outcomes (normal, low density, osteoporosis) using the Chi-square test. Meanwhile, differences in serum levels of inflammatory biomarkers between both groups will be measured using t-test. In order to account for missing data, survival analyses will be conducted while implementing the “last-observation carried forward” approach. Such data will be presented in Kaplan–Meier curves, showing the numbers of patients analyzed at each assessment timepoint. A *p*-value of less than 0.05 will indicate statistical significance.

### Ethics and dissemination

#### Informed consent

Before conducting activities related to the study, an investigator will verbally describe the information regarding the study and treatment medications to the patient, as well as provide written information that can be easily read and understood by the participant. An informed consent form with the subject's name and date will be obtained before allocation to treatment arms. The written informed consent will also be signed and dated by the investigator obtaining the informed consent. All patients will be informed that they can refuse to participate in the study and withdraw from the study at any time without affecting their medical care. Each patient will receive notification that information regarding their medical conditions will be collected and recorded and maintained in strict confidential manner and will only be used for research purposes.

#### Criteria for discontinuing allocated interventions

Patients who fail to complete the clinical protocol will be considered a dropout of the clinical study. Cases of attrition shall be recorded in detail, and the case report form (CRF) will be kept for future reference. Patients may withdraw spontaneously from the study at any time for any reason without consequences. An investigator may end the patient’s participation if they do not complete follow-up visits or comply with medication treatments. The study will be terminated if significant deviations occur in the design or implementation of the clinical study scheme which makes it difficult to evaluate drug effect. The study will be halted if ordered by the National Medical Products Administration or the researchers decide it is necessary to stop the trial.

#### Adverse Events (AEs)

All information regarding adverse events, whether mentioned by the patient, found by the investigator, or found through physical examination, laboratory examination, or other means, shall be recorded on the adverse events page of the case report form and shall be handled and reported in accordance with applicable regulations. The final clinical study report will include the adverse event reports.

The safety of all subjects receiving at least one dose will be evaluated, and all of the following events will be recorded: adverse events (AEs), serious adverse events (SAEs), death, stroke, discontinuation of medication for any reason, laboratory abnormalities, changes in vital signs, changes in physical examination, etc. Adverse events are to be recorded from the beginning of randomization through the 12 months of enrollment or until the last follow-up. If an adverse event occurs, the adverse event will be treated as appropriate until resolution.

If an adverse event occurs to a subject during the study period, it will be up to the subject's physician to determine whether the event was associated with the study and was indeed caused by the study drug. The patient will receive prompt and appropriate treatment under the care of their doctor. Each patient in the clinical study will be insured and all expenses incurred during participation in the study will be reimbursed.

#### Confidentiality

Safeguards will be taken to maintain strict confidentiality of all patients and specific research identification codes will be used for each participant. The principal investigator will safeguard all research data after completion of the study in accordance to research guidelines. The research team will have access to the identification codes only during the study. No patient identification information will be reported in the publication.

#### Data management

The case report form (CRF) will be completed by the investigator at the time of initiation of treatment and each follow-up visit. All case report forms will be collected by the electronic data acquisition system (EDC) and reviewed by the clinical supervisor. The forms will then be submitted to the data manager for compilation of data.

The CRF is designed to ensure all data specified in the test protocol are collected to meet the requirements of statistical analysis. Guidelines for filling in the corresponding CRF will be established and followed by each investigator to maintain uniformity. Establish logical verification according to the design instructions of the database, and put it into use after User Acceptance Testing (UAT) is qualified.

Data administrators, clinical inspectors, medical specialists, and statisticians will conduct data verification in accordance with the data verification plan (DVP), data verification content, methods, and verification requirements. A process will be formulated in regards to database locking, the responsible person, and the SOP file of execution, and to specify the conditions and procedures of unlocking and re-locking after database usage.

Development of guidelines for the file format, exported contents (database, variable name, and variable value code), submission procedure, and transmission medium of data exported and transmitted. The transmission medium shall comply with the requirements of national laws and regulations of regulatory authorities. The data management plan shall determine the quality control items, quality control methods (such as quality control frequency, sample selection methods, and sample size, etc.), quality requirements and standards, and remedial measures for the failure to meet the expected quality standards.

#### Dissemination of data

The results of this research study will be published in a peer-reviewed international medical journal. Both positive and negative results will be published. The principal investigator will provide, upon reasonable request, the datasets used during this study in accordance to the data transfer guidelines of this protocol. Participants will be provided a layman summary of the results from this clinical study.

## Discussion

To date, there are no clear data regarding the efficacy of alendronate sodium vitamin D3 regarding the stability of the knee joint, the progression and severity of OA, knee pain, and quality of life among patients with early knee osteoarthritis. Therefore, this randomized placebo-controlled trial is designed to provide high-quality evidence regarding alendronate’s efficacy in this patient population.

### Strengths and limitations

#### Strengths:

- The study is a randomized, placebo-controlled, double-blinded study.
- This study evaluates a novel treatment medication for osteoarthritis.
- This study will provide data on cartilage structural changes, as well as quality of life and pain changes related to the medication use.

#### Limitations:

- Follow-up for knee replacement will be over a period of 2 years which may not be long enough in patients with early osteoarthritis.
- Although this study will provide guidance regarding the effects of alendronate, we will not be able to determine the ideal treatment length for osteoarthritis.

## Supplementary Information

**Additional file 1: Supplementary Figure 1.** The different features incorporated within the WORMS scale.

**Additional file 2: Supplementary Figure 2.** The fourteen articular-surface regions of each feature within the WORMS scale.

**Additional file 3: Supplementary Table 1.** The sequence and parameters of the MRI unit used in our center.

## Data Availability

All data generated or analyzed during this study are included in this published article and its supplementary information files.

## Abbreviations

OA: Osteoarthritis
WORMS: Whole-Organ Magnetic Resonance Imaging Score
KOA: Knee osteoarthritis
DMOADs: Disease-modifying drugs
OAI: Osteoarthritis Initiative
JSN: Joint space narrowing
