# Supplementary figures and images for: Effect of alendronate sodium plus vitamin D3 tablets on knee joint structure and osteoarthritis pain: a multi-center, randomized, double-blind, placebo-controlled study protocol
Source: BMC Musculoskelet Disord. 2022 Jun 17;23:584. doi: 10.1186/s12891-022-05521-4 (PMC9205115; doi:10.1186/s12891-022-05521-4)

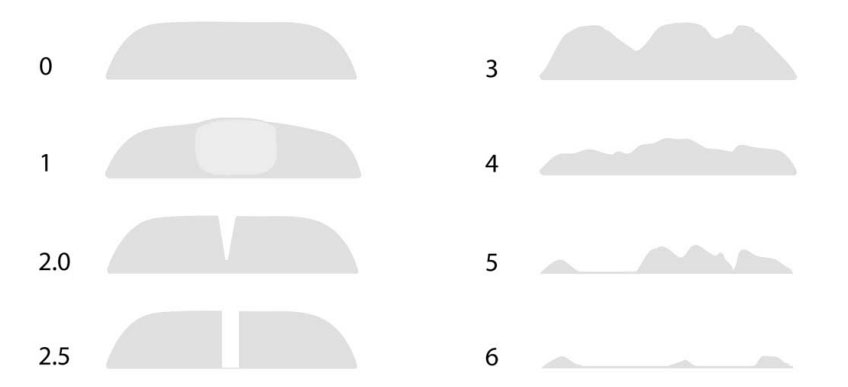

Supplement: Supplementary file 1 — Additional file 1: Supplementary Figure 1. The different features incorporated within the WORMS scale. [file 12891_2022_5521_MOESM1_ESM.jpg]

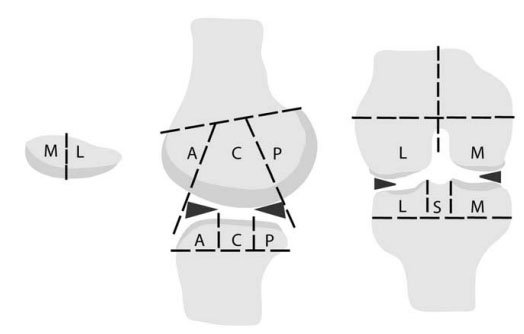

Supplement: Supplementary file 2 — Additional file 2: Supplementary Figure 2. The fourteen articular-surface regions of each feature within the WORMS scale. [file 12891_2022_5521_MOESM2_ESM.jpg]
