# Supplementary material for: Effect of alendronate sodium plus vitamin D3 tablets on knee joint structure and osteoarthritis pain: a multi-center, randomized, double-blind, placebo-controlled study protocol
Source: BMC Musculoskelet Disord. 2022 Jun 17;23:584. doi: 10.1186/s12891-022-05521-4 (PMC9205115; doi:10.1186/s12891-022-05521-4)
Supplement: Supplementary file 3 — Additional file 3: Supplementary Table 1. The sequence and parameters of the MRI unit used in our center. [file 12891_2022_5521_MOESM3_ESM.docx]

**Supplementary Table.** Description of the sequences and parameters of the MRI unit at the study center

| **Machine and coil** | **T1-weighted sagittal** | **Proton density-weighted sagittal** |
| --- | --- | --- |
| 1.5 T whole-body MR unit (Siemens, Aera) using a dedicated 15-channel transmit-receive knee coil | T1-weighted fat-saturated 3D gradient-recalled acquisition; flip angle 30 degrees, repetition time 14.7 msec; echo time 6.74 msec; field of view 16 cm; 448 × 448 matrix; 1 excitation; slice thickness 1.5 mm | Proton density fat-saturated 2D fast spin echo sequence; flip angle 180 degrees; repetition time 3200 msec; echo time 39 msec; field of view 16 cm; 320 × 320 matrix; 1 excitation; slice thickness 3 mm |
